# Detecting language network alterations in mild cognitive impairment using task‐based fMRI and resting‐state fMRI: A comparative study

**DOI:** 10.1002/brb3.3518

**Published:** 2024-05-02

**Authors:** Kerem Kemik, Emel Ada, Berrin Çavuşoğlu, Cansu Aykaç, Derya Durusu Emek Savaş, Görsev Yener

**Affiliations:** ^1^ Department of Neuroscience, Institute of Health Sciences Dokuz Eylül University Izmir Turkey; ^2^ Department of Radiology Dokuz Eylül University Medicine Faculty Izmir Turkey; ^3^ Department of Medical Physics, Institute of Health Sciences Dokuz Eylül University Izmir Turkey; ^4^ Department of Psychology, Faculty of Literature Dokuz Eylül University Izmir Turkey; ^5^ Department of Neurology, Faculty of Medicine Izmir University of Economics İzmir Turkey

**Keywords:** independent component analysis, language network, mild cognitive impairment, resting‐state fMRI, task‐based fMRIi

## Abstract

**Objective:**

The objective of this study was to investigate the functional changes associated with mild cognitive impairment (MCI) using independent component analysis (ICA) with the word generation task functional magnetic resonance imaging (fMRI) and resting‐state fMRI.

**Methods:**

In this study 17 patients with MCI and age and education‐matched 17 healthy individuals as control group are investigated. All participants underwent resting‐state fMRI and task‐based fMRI while performing the word generation task. ICA was used to identify the appropriate independent components (ICs) and their associated networks. The Dice Coefficient method was used to determine the relevance of the ICs to the networks of interest.

**Results:**

IC‐14 was found relevant to language network in both resting‐state and task‐based fMRI, IC‐4 to visual, and IC‐28 to dorsal attention network (DAN) in word generation task‐based fMRI by Sorento‐Dice Coefficient. ICA showed increased activation in language network, which had a larger voxel size in resting‐state functional MRI than word generation task‐based fMRI in the bilateral lingual gyrus. Right temporo‐occipital fusiform cortex, right hippocampus, and right thalamus were also activated in the task‐based fMRI. Decreased activation was found in DAN and visual network MCI patients in word generation task‐based fMRI.

**Conclusion:**

Task‐based fMRI and ICA are more sophisticated and reliable tools in evaluation cognitive impairments in language processing. Our findings support the neural mechanisms of the cognitive impairments in MCI.

## Graphical abstract

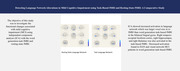

## 1 INTRODUCTION

Alzheimer's disease (AD) is a common degenerative disease characterized by cognitive and memory impairment (Petersen, 2000). Mild cognitive impairment (MCI) is a transitional stage between normal aging and dementia, in which individuals exhibit significant deficits in one or more cognitive functions but can still maintain their functional lifestyle and have not yet developed dementia. However, more than half of MCI patients develop dementia within 5 years (Gauthier et al., 2006), making it essential to investigate the contributing factors to determine this conversion. AD is primarily caused by the accumulation of beta‐amyloid and tau protein and formation of plaques and tangles in the brain, which begins many years before clinical symptoms start. As a multifactorial disease, AD has been shown to cause network and neuron activity changes in EEG and functional magnetic resonance imaging (fMRI) studies (Peter et al., 2014).

Despite the prevalence of AD dementia and MCI, countless research has examined the language and cognitive deficits associated with neurodegenerative diseases (Li et al., 2015; Paek et al., 2020; Pereira et al., 2018; Pulido et al., 2020). Fluency tasks have been utilized to measure cognitive and lexical capabilities, with three main types of tasks used: action, letter, and category fluency tasks, which show frontostriatal and frontotemporal activities. These functions are affected in damaged areas, and each patient exhibits different rates of progression (Nutter‐Upham et al., 2008). Recent research shows that one of the first affected areas of the brain is in language network. Especially in resting‐state functional MRI (rs‐fMRI), significantly increased activity has been shown in MCI patients (Pistono et al., 2021).

Of these fluency tasks, studies have found that letter fluency tasks have the potential to detect and predict cognitive declines due to their high demand for cognitive processes, including semantic memory, strategic search, inhibition, and working memory (Belleville et al., 2017) Furthermore, letter fluency normative studies have indicated that task performance can be effected by specific cultural or country‐related factors. Letter fluency tasks found to be able to identify cognitive deficits in AD and major depressive disorder (St‐Hilaire et al., 2016).

Fluency tasks, such as action, letter, and category, have gained popularity in fMRI research due to their ease of application in MRI machines and their ability to reveal activity changes in patients with AD and other neurodegenerative diseases in specific areas (Boksman et al., 2005). During the letter fluency task commonly generate words from a given letter. In the word generation task, participants are required to continue generating words from the last letter of the previous word, adding reading and switching steps to the task and allowing for the identification of more frontotemporal language connections (Bak & Hodges, 2003).

In recent studies on dementia and MCI, fluency tasks utilize to explore cognitive deficits and neurofunctional changes during functional analysis (Chételat et al., 2009). These studies investigate the brain's functional connectivity, uncovering nonanatomical connections or correlations between seeds or regions of interest (ROIs) (Bullmore & Sporns, 2009). Functional connectivity describes synchronized activity patterns between different brain regions. For instance, recent rs‐fMRI studies have revealed disruptions in the default mode network and alterations in the salience network in individuals with MCI and AD (Badhwar et al., 2017; Wang et al., 2012; Zhou et al., 2012). These findings accentuate the importance of functional neuroimaging techniques to comprehensively characterize cognitive impairments and inform diagnostic and therapeutic strategies (Chételat et al., 2009). Various methods have been developed over the years to determine connectivity analysis, all of which aim to define the relationship between brain regions beyond structural connections (Du et al., 2018; Rosazza et al., 2012; Sporns, 2018).

Independent component analysis (ICA) has emerged as a promising approach for analyzing group task‐based fMRI data, offering several advantages over traditional ROI‐based methods. By decomposing fMRI data into independent components (ICs), ICA provides a comprehensive exploration of functional connectivity, capturing both well‐established and novel functional networks. This unbiased approach enables the extraction of subject‐level components that can be aggregated across participants, facilitating robust group‐level analyses. Through ICA, shared network architectures underlying task‐related functional connectivity can be identified, enhancing our understanding of cognitive processes.

One notable advantage of ICA is its ability to separate signal from noise, allowing for a more accurate representation of underlying brain activity. Additionally, ICA is well‐suited for examining complex interactions between brain regions during task‐based fMRI experiments, shedding light on the intricate dynamics of cognitive processes. As a result, the increasing adoption of ICA as the preferred method for investigating functional connectivity in group task fMRI studies promises for advancing our understanding of cognitive processes and neurological disorders (Arfanakis et al., 2000; Calhoun & Adali, 2006; Rosazza et al., 2012).

The application of ICA in the analysis of group task‐based fMRI data offers substantial benefits, allowing for a comprehensive exploration of functional connectivity and facilitating the identification of shared network architectures. By leveraging the strengths of ICA, researchers can unravel the complexities of cognitive processes and gain valuable insights into the pathophysiology of neurological disorders.

In this study, we performed fMRI using the word generation task and rs‐fMRI on individuals with MCI and healthy controls (HCs) to evaluate the functional changes associated with MCI. We aimed to investigate the network changes of MCI patients compared to healthy subjects by conducting functional connectivity analyses on task‐based fMRI data and rs‐fMRI. We hypothesized that task‐based fMRI of the specific MCI groups compared with rs‐fMRI data will help better understand the nature of cognitive decline, and task‐based fMRI can reveal distinct alterations in the language network of individuals with MCI compared to HCs. Specifically, decreased functional connectivity within the language network during the word generation task in MCI patients is indicative of impaired language processing. Additionally, we anticipate compensatory activity in other brain regions, as MCI patients may recruit alternative neural pathways to maintain cognitive function. Furthermore, we predict that rs‐fMRI will also detect changes in the language network of MCI patients. Overall, our hypothesis suggests that both task‐based and rs‐fMRI analyses will provide valuable understandings into the neural mechanisms underlying cognitive impairments in MCI, contributing to the diagnosis and understanding of this condition. According to our knowledge, this is the first study that uses ICA for connectivity analysis in MCI patients with language task in fMRI. Furthermore, we predict that rs‐fMRI will also detect changes in the language network of MCI patients, even though to a lesser extent compared to task‐based fMRI. Overall, our hypothesis suggests that both task‐based and resting‐state fMRI analyses will provide valuable understanding into the neural mechanisms underlying cognitive impairments in MCI, contributing to the diagnosis and understanding of this condition.

## 2 METHODS

### 2.1 Participants

At the beginning, 20 patients with MCI and 20 demographically matched healthy controls (HCs) were recruited in the study. But six participants were excluded from the analysis, three of them due to poor image quality and the other three due to inability to perform the word tasks. The remaining 17 MCI and 17 HC were included in further analysis. The study protocol was approved by the ethics committee of the university, and all participants provided written informed consent prior to voluntary participation in the study.

MCI patients were recruited from the University Faculty of Medicine, Department of Neurology, Dementia Outpatient Clinic, and HCs were recruited from various community sources (Table 1).

**TABLE 1:** Sociodemographic characteristics of study participants.

	Healthy controls	MCI	*p*
Sex (M/F)	6/11	9/8	.394
Age (mean)	69.58	68.94	.786
Education (mean)	10.76	12.05	.540
MMSE scores	29.3 ± 0.75	25.5 ± 1.3	<.001^a^

*Note*: Values are presented as mean or number of participants.

Abbreviations: F: female; HC: healthy control; M: male; MCI: mild cognitive impairment; MMSE; Mini–Mental State Examination.

No significant differences were observed between groups for any of the sociodemographic factors.

The inclusion criteria for HCs were no self‐reported cognitive complaints; no history of cognitive deficits and/or neurological abnormality (Mini‐Mental State Examination (MMSE) score ≥27).

The NIA‐AA diagnostic criteria were applied to MCI patients. The other inclusion criteria of the MCI patients were as follows: (1) clinical dementia rating score of 0.5; (2) memory complaints by the patient; (3) memory impairment defined with performances ≥1.5 standard deviation below for age‐ and education‐matched controls in a battery of neuropsychological tests; (4) preserved daily life functionality (Bell‐McGinty et al., 2005; Li et al., 2015).

The exclusion criteria of the MCI patients were as follows: (1) actual participation in a clinical trial using disease‐modifying drugs; (2) systematic use of antidepressant drugs with anticholinergic side effects. The exclusion criteria for all participants were as following: (1) history of psychiatric and/or neurological including evidence of depression as demonstrated by Yesavage Geriatric Depression Scale scores higher than 13; (2) history of drug and/or alcohol misuse and severe head injury; (3) presence of nonstabilized medical illnesses; (4) chronic use of analgesics, narcotics, sedatives, neuroleptics, or hypnotics and/or cognitive enhancers including acetylcholinesterase inhibitors; (5) presence of a hydrocephalus, brain tumor, or vascular brain lesions.

### 2.2 Neuropsychological measures

All participants underwent comprehensive neuropsychological assessment by trained examiners. MMSE (Gungen et al., 2002), Clinical Dementia Staging Scale clock drawing test, and Geriatric Depression Scale were administered to all participants to assess cognitive status, dementia staging, executive function, and mood symptoms, respectively.

### 2.3 Image acquisition

The MRI scans were performed using a 1.5 T MR Intera Achieva device (Philips Medical Systems, Best, The Netherlands). Using SENSE‐Head‐8 coil, anatomical T1‐weighted inversion‐recovery scan was acquired as reference images with the following parameters: TR: 2494 ms, TE: 15 ms, flip angle: 90°, ETL: 5, matrix: 512 × 512, slice thickness: 4 mm. For task‐based fMRI and rs‐fMRI, T2*‐weighted gradient echo‐planar images were acquired with the following parameters: TR: 3000 ms, TE: 50 ms, flip angle: 90, FOV: 230 mm, RFOV: 100%, slice thickness: 4 mm, gap: 0 mm, matrix scan: 64 × 64, ETL: 48, NA: 1 and approximately 28 slices. A total of 200 dynamic series were obtained with 8 passive and 12 active periods repeated 10 times, resulting in a total scan time of 10 min. In resting state fMRI acquisitions, all 200 dynamics acquired. All participants performed the Word Generation Task using the Telemed software and equipment. Before to the fMRI examination, participants were familiarized with the task and trained with the same set of words to ensure consistency. During the task, participants were presented with a word on the screen and were asked to generate a new word starting with the last letter of the presented word. The task consisted of ten active periods, separated by passive periods.

### 2.4 Word generation task

Prior to the fMRI session, the task was demonstrated and taught to each participant using identical words. The general common word list for the Turkish language was used for ease of understanding. Patients receive this example in the same order: you will see a word on the screen, and while you are seeing a word; starts with its last letter and continue with your words last letter. For example; “computer” seen on the screen, next word starts with an R letter then continue with word beginning last letter of previous word. You will generate as much as you can until seeing the sign “Please Stop Now.” Then you will continue with new word. During the task, the words are seen on the screen through a mirror that was attached to the head coil. A new word was presented on the screen randomly during each active state, allowing participants approximately 3 s to read and found of new words. The fMRI sequence consisted of 10 dynamic series of the 12 active and 8 passives. The fMRI data were acquired during the active states of the task.

### 2.5 fMRI analysis

In this study, CONN (version 18.b, RRID:SCR_009550) (Du et al., 2018) and SPM (version 12.7771, RRID:SCR_007037) (Nichols et al., 2011) used software packages to preprocess and analyze the functional and anatomical data. CONN standard preprocessing pipeline (Nieto‐Castanon, 2020) was utilized for preprocessing, consisting of several sequential steps. Initially, the functional data underwent realignment using the SPM realign & unwarp procedure (Andersson et al., 2001) to correct for motion and magnetic susceptibility interactions. This involved coregistering all scans to a reference image, which was the first scan of the first session, using a least squares approach and a six‐parameter (rigid body) transformation (Friston et al., 1995). Subsequently, the temporal misalignment between different slices of the functional data, acquired in descending order, was corrected using the SPM slice‐timing correction procedure (Henson et al., 1999; Sladky et al., 2011), Sinc temporal interpolation was employed in this procedure to resample each slice BOLD time series to a common mid‐acquisition time. Outlier scans were identified using the artifact detection tools (Whitfield‐Gabrieli et al., 2011), considering scans with framewise displacement exceeding 0.9 mm or global BOLD signal changes beyond 5 standard deviations (Nieto‐Castanon, 2022; Power et al., 2014). A reference BOLD image for each subject was computed by averaging all scans, excluding the outliers.

Normalization of both functional and anatomical data was accomplished using the SPM unified segmentation and normalization algorithm (Ashburner, 2007; Ashburner & Friston, 2005) employing the default IXI‐549 tissue probability map template. This process involved direct normalization of the functional and anatomical data to standard MNI space, followed by segmentation into gray matter, white matter, and CSF tissue classes. The data were then resampled to 4 mm isotropic voxels.

Furthermore, the functional data underwent spatial smoothing using a Gaussian kernel with a full width at half maximum of 8 mm. The Gaussian kernel is used for getting a better signal/noise ratio and it is recommended about two times the slice thickness.

Additionally, a standard denoising pipeline (Nieto‐Castanon, 2020) was applied to the functional data. This pipeline included the regression of potential confounding effects, such as white matter time series (5 CompCor noise components), CSF time series (5 CompCor noise components), motion parameters and their first‐order derivatives (12 factors) (Friston et al., 1996), outlier scans (below 91 factors) (Power et al., 2014), session and task effects and their first‐order derivatives (6 factors), and linear trends (2 factors) within each functional run. Following this, bandpass frequency filtering of the BOLD time series (Hallquist et al., 2013) limits the frequencies between 0.008 and 0.09 Hz. CompCor noise components within white matter and CSF were estimated by computing the average BOLD signal and extracting the largest principal components orthogonal to the BOLD average, motion parameters, and outlier scans within each subject's eroded segmentation masks (Behzadi et al., 2007; Chai et al., 2012).

*Group‐level independent component analyses* (group‐ICA) (Calhoun et al., 2001) were conducted to estimate 40 temporally coherent networks from the fMRI data, combined across all subjects and conditions. The BOLD signal from every timepoint and voxel in the brain was concatenated along the temporal dimension across subjects and conditions. A subject‐level dimensionality reduction step involved performing a singular value decomposition (SVD) of the *z*‐score normalized BOLD signal with 64 components separately for each subject and condition. Further dimensionality reduction was achieved using a SVD (group‐level) with 40 components. The resulting components were then subjected to a fast‐ICA fixed‐point algorithm (Hyvarinen, 1999) with a hyperbolic tangent (G1) contrast function used to identify spatially independent group‐level networks from the resulting components. Last, GICA3 back‐projection (Erhardt et al., 2011) was applied to compute ICA maps associated with these networks separately for each individual subject and condition.

Group‐level analyses were performed using a general linear model (GLM) (Nieto‐Castanon, 2020). For each individual voxel, a separate GLM was estimated, with first‐level connectivity measures at this voxel as dependent variables (one independent sample per subject and one measurement per task or experimental condition, if applicable), and groups or other subject‐level identifiers as independent variables. Voxel‐level hypotheses were evaluated using multivariate parametric statistics with random‐effects across subjects and sample covariance estimation across multiple measurements. Inferences were performed at the cluster level, considering groups of contiguous voxels. Cluster‐level inferences were based on parametric statistics derived from Gaussian Random Field theory (Nieto‐Castanon, 2020; Worsley et al., 1996). The results were thresholded using a combination of a cluster‐forming *p* < .001 voxel‐level threshold and a family‐wise corrected *p*‐FDR < .05 cluster‐size threshold (Chumbley et al., 2010).

The significance of these components on neural networks was determined by assessing the spatial overlap with visually selected best‐matched IC using the Dice Similarity Coefficient or Sørensen‐Dice Index (explanation added as Supportion Information) (Cocchi et al., 2012; Sorg et al., 2007; Zou et al., 2004). Dice Coefficient is a tool that is used to determine spatial overlay on component with the given networks.

### 2.6 Statistical analysis

Statistical analyses were conducted using SPSS 24, including Shapiro–Wilk, Mann–Whitney *U* tests, and chi‐square tests. The protocol was implemented with a contrast vector of [(Petersen, 2000),‐Petersen, 2000),0] for Control > MCI Group, with age included as a covariate. Consequently, in the color map ranging from blue to red, regions highlighted in red denote decreased activity in the MCI group, while those in blue signify increased activity, owing to the negative inversion. For statistical analysis, a 3 × 2 mixed ANOVA interaction was utilized for comparison (Nieto‐Castanon, 2022).

## 3 RESULTS

### 3.1 Demographic and clinical variables characteristics of the participants

The demographic and clinical characteristics are shown in Table 1. The MCI and HC groups were matched based on age, sex, and education (Table 1). No significant differences were found between the two groups in terms of age, education, or sex (*p* > .05 for all comparisons). MCI presented poorer scores on MMSE than HCs (*p* < .001).

### 3.2 Selection of the IC's

**FIGURE 1:**
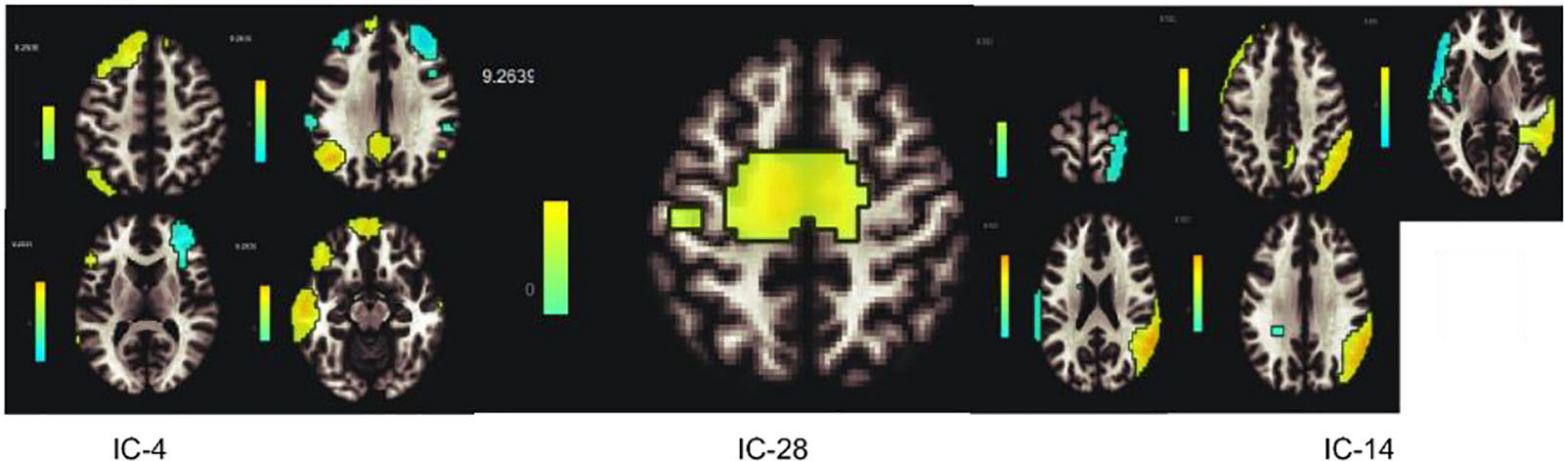
Spatial maps of selected independent components (IC's).

The analysis showed that IC‐4, IC‐14, and IC‐28 were the best matches for the selected ICs, which they are visual, language, and dorsal attention networks (DANs) respectively, determined by the Sorento‐Dice Coefficient (Figure 1). Dice Coefficient table, which shows the best matches of the IC's, is added as supplementary.

### 3.3 Resting state functional connectivity

**FIGURE 2:**
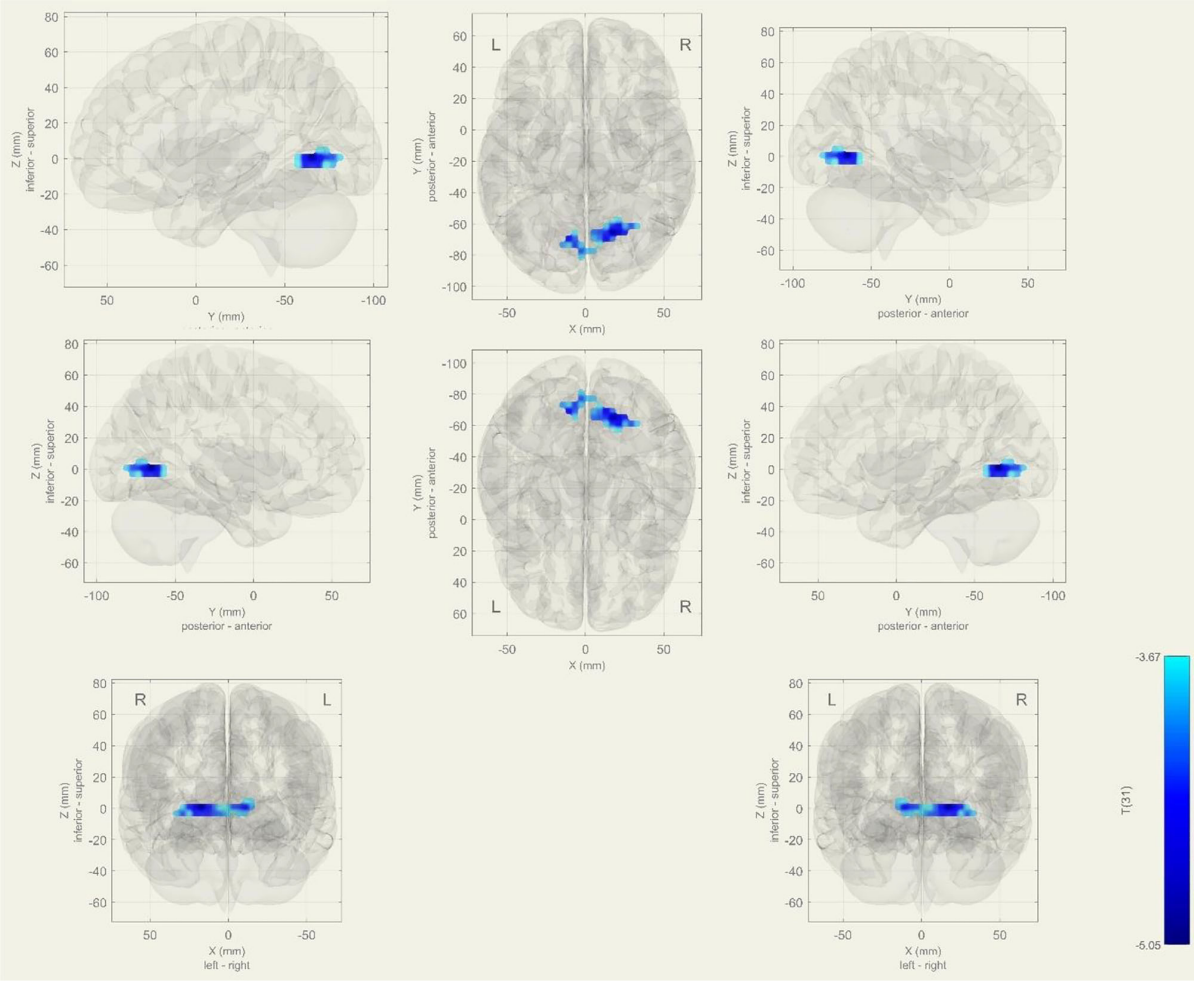
In resting state, bilateral lingual gyrus and intercalcarine cortexes observed increased functional connectivity at language network in mild cognitive impairment (MCI) group (*p*‐FDR = 0.0003). As a contrast vector effect, blue areas shows decreased activity in the MCI group, on the contrary; red areas shows increased activity in MCI.

During rs‐fMRI, IC‐14 was the only matched component in the language network to show significant results as determined by the Sorento‐Dice Coefficient. The MCI group exhibited increased functional connectivity in bilateral lingual and intercalcarin cortices in the language network (*p*‐FDR = .0003) (Figure 2) (Table 2).

**TABLE 2:** Results of independent component analysis (ICA) for Word Generation Task and Resting‐State functional magnetic resonance imaging (fMRI).

	Coordinates (*X*,*Y*,*Z*)	TVS	*p*‐FDR	Network	Area	%ROI	Voxel size
**rs‐fMRI**	+18 −66 +00	392	.0003	Language	Right lingual gyrus Left lingual gyrus. Left intercalcarine cortex Right intercalcarine cortex	12 6 3 1	206 84 18 4

Abbreviations: DAN, Dorsal Attention Network; FDR, False discovery rate; ROI, regions of interest; rs‐fMRI, resting state functional magnetic resonance imaging; TO, Temporooccipital; TVS: Total Voxel Size.

### 3.4 Word generation task functional connectivity

During the word generation task, fMRI, IC 4, IC 14, and IC 28 were matched component as visual, language, and DANs, respectively, to show significant results as determined by the Sorento‐Dice Coefficient (Table 3). The results provide coordinates, voxel size, *p*‐FDR rate, the network involved, the area given by the MNI atlas, the percentage of total ROI area, and voxel size.

**TABLE 3:** Word Generation Task independent component analysis (ICA) results.

	Coordinates (*X*,*Y*,*Z*)	TVS	*p*‐FDR	Network	Area	%ROI	Voxel size
**Word generation task**	+30 −10 +64	280	.0106	Visual	Precentral gyrus Right Superior frontal gyrus right Middle frontal gyrus right	5 1 1	206 40 26
	‐06 −58 −12 +26 −50 −16	352 288	.0003 .0008	Language	Left lingual gyrus. Right TO‐fusiform cortex Right lingual gyrus Right hippocampus Right thalamus	9 14 5 2 0	131 111 78 12 1
	+02 +14 +56	168	.045	DAN	Right superior frontal syrus Left superior frontal gyrus Right paracingulate gyrus	4 1 0	111 24 2

Abbreviations: DAN, dorsal attention network; FDR, false discovery rate; ROI, regions of interest; TO, temporooccipital; TVS, total voxel size.

**FIGURE 3:**
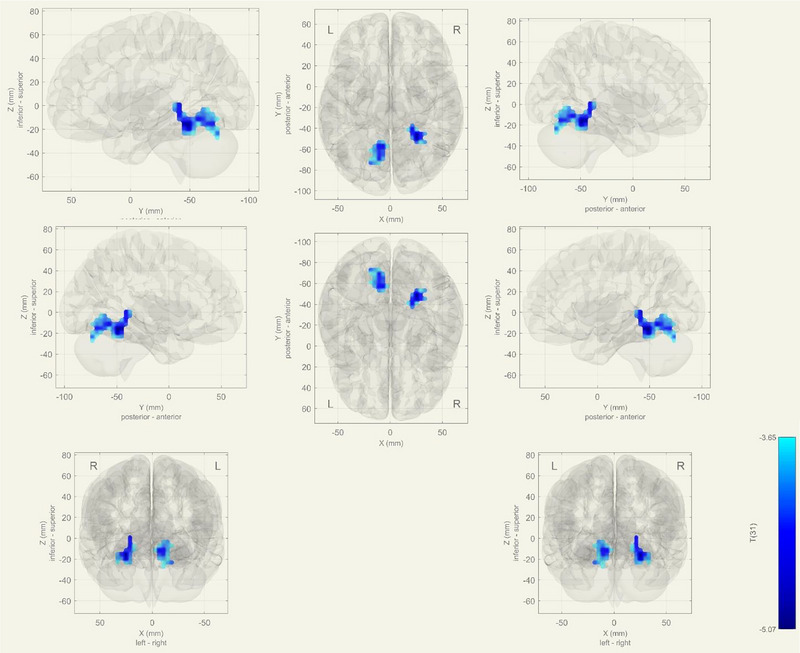
During task, increased functional connectivity seen at bilateral lingual gyrus, Right temporooccipital cortex and Right hippocampus and thalamus at language network in mild cognitive impairment (MCI) group (*p*‐FDR = 0.0016). As a contrast vector effect, blue areas show decreased activity in the MCI group, on the contrary; red areas shows increased activity in MCI.

In MCI group, increased functional connectivity was detected in bilateral lingual gyrus, and right temporo‐occipital cortex, r hippocampus, and r thalamus, which are in the language network (*p*‐FDR = .0016) (Figure 3).

**FIGURE 4:**
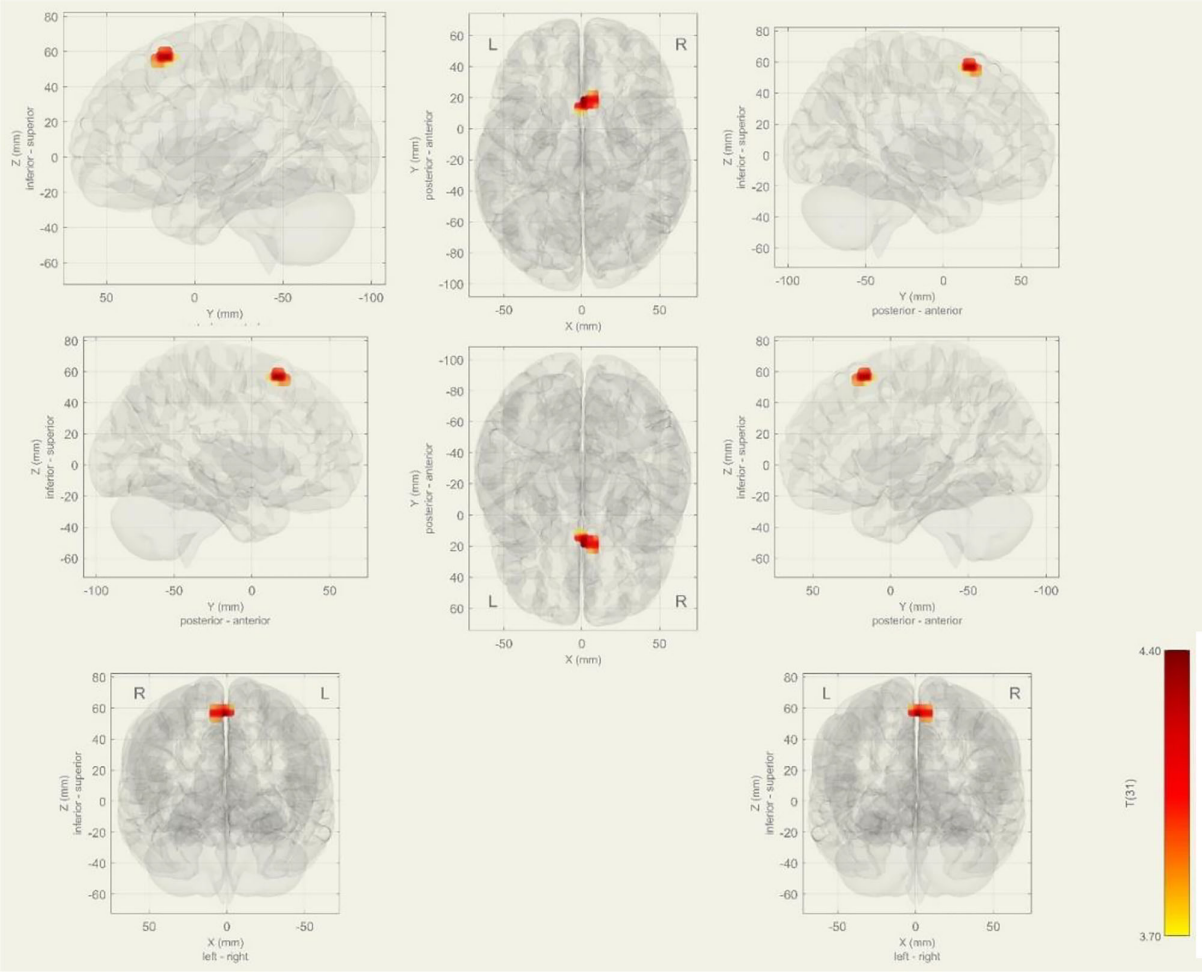
During task, decreased functional connectivity observed at bilateral superior frontal gyrus and paracingulate gyrus at Dorsal Attention network in mild cognitive impairment (MCI) group (*p*‐FDR = 0.0447). As a contrast vector effect, blue areas show decreased activity in the MCI group, on the contrary; red areas shows increased activity in MCI.

Significant decreased functional connectivity in the right precentral gyrus, right superior frontal gyrus, and right middle frontal gyrus in the DAN (*p*‐FDR = .0106) (Figure 4).

**FIGURE 5:**
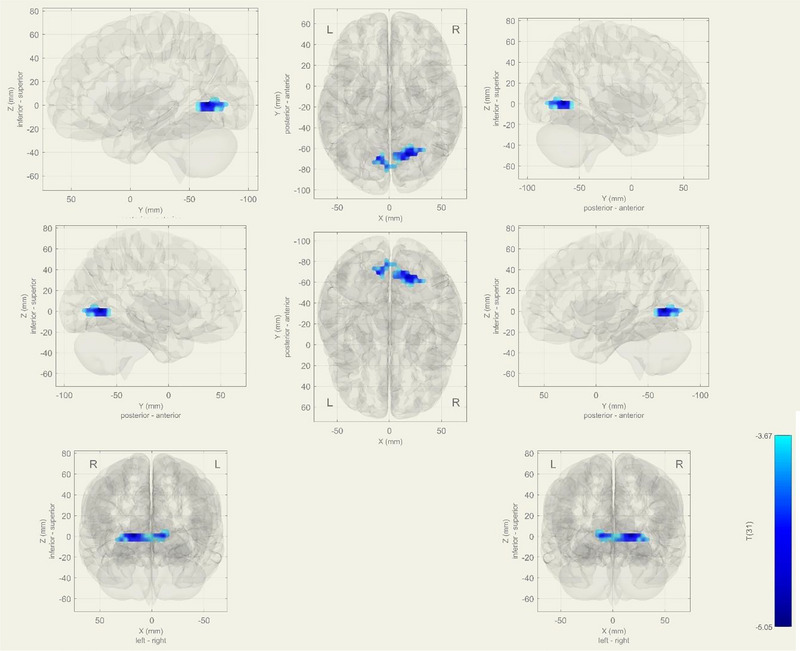
During task, decreased functional connectivity observed at bilateral Superior frontal gyrus and paracingulate gyrus at Dnetwork in mild cognitive impairment (MCI) group (*p*‐FDR = 0.0447). As a contrast vector effect, blue areas show decreased activity in the MCI group, on the contrary; red areas show increased activity in MCI.

In the visual network, the MCI group exhibited decreased functional connectivity in bilateral superior frontal gyrus and paracingulate gyrus in the DAN (*p*‐FDR = .0447) (Figure 5).

The results provide coordinates, voxel size, *p*‐FDR rate, the network involved, the area given by the MNI atlas, the percentage of total ROI area, and voxel size.

## 4 DISCUSSION

We investigate the functional changes on neural networks associated with MCI using ICA in both word generation task fMRI and resting‐state fMRI.

In this study, we found bigger voxel size in the resting state fMRI in the language network, particularly the lingual gyrus, it was more active in the resting state fMRI than word generation task means increased activity in the lingual gyrus indicating language network impaired in MCI patients. This can be thought as a compensatory mechanism to maintain the connectivity structure of neurons. However, it can also lead to the production of more tau proteins, which can be harmful to connected neurons (Hillary & Grafman, 2017). However, compensatory activity was also observed in the right temporal occipital fusiform cortex, right hippocampus, and right thalamus during the word generation task too.

Our findings are not consistent with previous studies that have demonstrated impaired connectivity within the language network in MCI patients (Kiely et al., 2014; Lin et al., 2011; Pistono et al., 2021; Xu et al., 2016). For instance, studies by Xu et al. (2016) and Lin et al. (2011) have reported decreased connectivity within various regions of the language network in aMCI patients, including the left inferior frontal gyrus, the left middle frontal gyrus, the left superior temporal gyrus, and the left middle temporal gyrus. However, contrary to these findings, our study revealed increased activity within the language network, particularly in the lingual gyrus. This discrepancy suggests potential variability in language network alterations across MCI patients and underscores the complexity of neural changes associated with this condition. On the other hand, our results contribute to the evidence regarding the complex nature of language network alterations in MCI, highlighting the need for further investigation into their role as potential indicators of cognitive impairment. Our findings are parallel with previous studies utilizing a functional near‐infrared spectroscopy (fNIRS) methodology in the literature. For instance, a study utilizing fNIRS reported hyperconnectivity between hemispheres during the resting state and language task. Additionally, these results highlight the importance of considering both resting‐state and task‐based functional connectivity to gain a comprehensive understanding of brain network dynamics in cognitive impairment (Nguyen et al., 2019; Paek et al., 2020).

In addition to the language network, our results also revealed differences in the visual and DANs between the MCI and HC groups. Specifically, we observed decreased activity in the right precentral gyrus, right superior frontal gyrus, and middle frontal gyrus of the visual network in the aMCI group. We observed decreased functional connectivity in the DAN during the word generation task, in the right superior frontal gyrus, left superior frontal gyrus, and right precentral gyrus in the MCI group. Lin et al. also demonstrated decreased functional connectivity within the visual network in MCI patients (Figure 2) (Lin et al., 2011). Another study reported decreased connectivity within the DAN in MCI patients, suggesting that impairments in this network (Yamasaki et al., 2019; Yamashita et al., 2019). The DAN is also a crucial network in MCI studies as it is one of the first impairments in this network may be an early sign of cognitive decline. This may be associated with the deprivation of neuron connectivity, which is affected by the disease (Soman et al., 2020). Differences can be dependent on the MCI patients’ stages.

The word generation task is a useful tool in defining the language network, as demonstrated by its ability to detect changes in critical areas relevant to MCI and dementia (Paek et al., 2020). In the present study, we observed decreased activity in the hippocampus and thalamus, calcarine, and frontal areas during the word generation task, indicating that these areas are significantly affected in MCI patients. Although the task may differ in its application, such as deriving non‐verb words or counting the produced words, studies have consistently shown activity changes in critical areas relevant to MCI and dementia, highlighting the diagnostic benefits of task‐based evaluations over resting‐state analysis (Paek et al., 2020).

### 4.1 Strengths and limitations

Based on the power analysis, a sample size of at least 17 participants in each group was sufficient to detect statistically significant differences in brain activation between the MCI and HC groups. But this sample size meets the minimum requirement for statistical power, larger sample sizes would increase the precision of the results and allow for more strong conclusions.

In terms of strengths, our study utilized task‐based fMRI to investigate language processing in MCI, providing valuable insights into the neural mechanisms underlying language deficits in this population. Additionally, our study included a well‐matched control group and applied rigorous statistical analyses to ensure the validity of our findings.

However, our study had several limitations. First, the small sample size may limit the generalizability of our findings to larger populations. Second, our study did not investigate language deficits in MCI patients with specific cognitive deficits, limiting the specificity of our results. Lastly, as with all cognitive tasks applied to MCI patients, individual differences in prognosis and disease severity may have affected our results, highlighting the need for future studies to address these limitations.

## 5 CONCLUSION

Our study demonstrates increased activity within the language network, particularly in the lingual gyrus, in individuals with MCI compared to HCs. These findings suggest a compensatory mechanism to maintain neuronal connectivity in MCI patients. Additionally, differences in the visual and DANs were observed between the MCI and HC groups. Our results highlight the importance of task‐based fMRI and resting‐state fMRI combined with ICA in understanding cognitive impairments in MCI and provide valuable insights into the neural mechanisms underlying language deficits in this population. This study can contribute to the diagnosis and prevention of MCI and AD by providing a better understanding of the functional changes in the brain associated with these conditions.

## AUTHOR CONTRIBUTIONS

**Kerem Kemik**: Conceptualization; investigation; funding acquisition; writing—original draft; methodology; validation; visualization; writing—review and editing; software; formal analysis; data curation; resources. **Emel Ada**: Conceptualization; investigation; funding acquisition; writing—original draft; writing—review and editing; visualization; validation; methodology; software; formal analysis; project administration; data curation; supervision; resources. **Berrin Çavuşoğlu**: Conceptualization; investigation; funding acquisition; writing—original draft; methodology; validation; writing—review and editing; supervision. **Cansu Aykaç**: Writing—original draft; data curation; resources; software; writing—review and editing; methodology; validation; visualization. **Derya Durusu Emek Savaş**: Conceptualization; investigation; funding acquisition; methodology; visualization; validation; supervision; data curation. **Görsev Yener**: Conceptualization; investigation; funding acquisition; writing—original draft; methodology; validation; visualization; writing—review and editing; software; formal analysis; project administration; data curation; supervision; resources.

## CONFLICT OF INTEREST STATEMENT

The authors declare that they have no financial or personal relationships that may have influenced the work presented in this manuscript. They have no conflicts of interest to disclose.

## FUNDING STATEMENT

Scientific Research Projects Coordination Unit of the Dokuz Eylül University, Grant number: 2014‐154.

### PEER REVIEW

The peer review history for this article is available at https://publons.com/publon/10.1002/brb3.3518.

## Supporting information

Supporting Information

## Data Availability

The data that support the findings of this study are available on request from the corresponding author. The data are not publicly available due to privacy or ethical restrictions.
