# Supplementary material for: Detecting language network alterations in mild cognitive impairment using task‐based fMRI and resting‐state fMRI: A comparative study
Source: Brain Behav. 2024 May 2;14(5):e3518. doi: 10.1002/brb3.3518 (PMC11066416; doi:10.1002/brb3.3518)
Supplement: Supplementary file 1 — Supporting Information [file BRB3-14-e3518-s001.docx]

Figure 1. Dice Coefficiency Map of the Indepentent Components


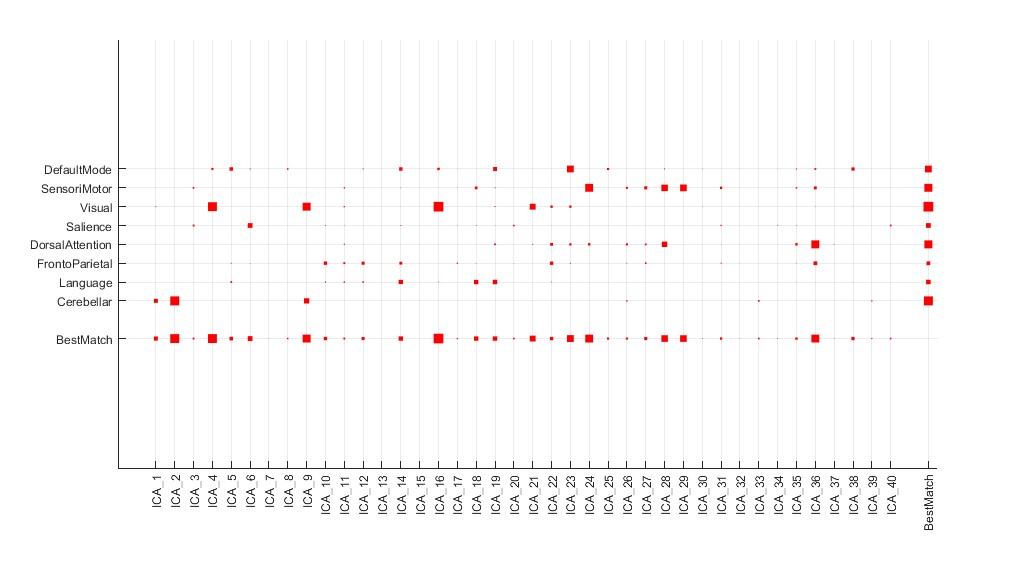


Best match means the IC coeeficiency is most with the selected network.

Dice Coefficiency:

The Dice coefficient, also known as the Sørensen-Dice coefficient, is a measure commonly used to quantify the spatial overlap between two binary images or regions of interest (ROIs). In the context of independent component analysis (ICA) in fMRI, the Dice coefficient can be used to assess the similarity or agreement between the spatial maps derived from ICA and anatomical or functional templates.

Specifically, when applying ICA to fMRI data, spatial maps representing independent components (ICs) are generated, each capturing a distinct pattern of brain activity. To evaluate the reliability or validity of these ICs, researchers often compare them to known brain regions or functional networks derived from prior anatomical or functional knowledge.

The Dice coefficient provides a quantitative measure of the spatial similarity between the IC spatial maps and the reference templates. It calculates the spatial overlap between the two images by dividing twice the number of voxels that are present in both images by the total number of voxels in both images combined. The resulting coefficient ranges from 0 to 1, with higher values indicating greater spatial agreement or overlap between the images.

In fMRI studies, researchers may use the Dice coefficient to assess the degree of correspondence between IC spatial maps and known functional networks or anatomical structures. A higher Dice coefficient suggests a stronger resemblance between the IC spatial map and the reference template, supporting the validity of the IC as representing a meaningful neural network or brain region.[1]

Figure 2. Dice Coefficiency Formula


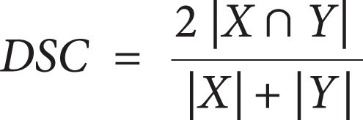
[2]

1. Nieto-Castanon, A., *Handbook of functional connectivity magnetic resonance imaging methods in CONN*. 2020: Hilbert Press.

2. Swerdlow, M., et al., *Simultaneous Segmentation and Classification of Pressure Injury Image Data Using Mask-R-CNN.* Computational and Mathematical Methods in Medicine, 2023. **2023**: p. 1-7.
